# Antimicrobial Activities of Chitosan Derivatives

**DOI:** 10.3390/pharmaceutics13101639

**Published:** 2021-10-08

**Authors:** Cristina Ardean, Corneliu Mircea Davidescu, Nicoleta Sorina Nemeş, Adina Negrea, Mihaela Ciopec, Narcis Duteanu, Petru Negrea, Daniel Duda-Seiman, Delia Muntean

**Affiliations:** 1 Faculty of Industrial Chemistry and Environmental Engineering, Politehnica University of Timişoara, 2 Piata Victoriei, 300006 Timisoara, Romania; cristina.ardean@student.upt.ro (C.A.); adina.negrea@upt.ro (A.N.); mihaela.ciopec@upt.ro (M.C.); petru.negrea@upt.ro (P.N.); 2 Renewable Energy Research Institute-ICER, University Politehnica of Timisoara, 138 Gavril Musicescu Street, 300501 Timisoara, Romania; corneliu.davidescu@upt.ro; 3 Department of Cardiology, Victor Babes University of Medicine and Pharmacy Timişoara, 2 Piata Eftimie Murgu, 300041 Timisoara, Romania; 4 Multidisciplinary Research Center on Antimicrobial Resistance, Department of Microbiology, Victor Babes University of Medicine and Pharmacy Timişoara, 2 Eftimie Murgu Square, 300041 Timisoara, Romania; muntean.delia@umft.ro

**Keywords:** chitosan, antibacterial activities, antifungal activities, functionalization by impregnation

## Abstract

Considering the challenge created by the development of bacterial and fungal strains resistant to multiple therapeutic variants, new molecules and materials with specific properties against these microorganisms can be synthesized, like those synthesized from biopolymers such as chitosan with improved antimicrobial activities. Antimicrobial activities of seven obtained materials were tested on four reference strains belonging to American Type Culture Collection. The best antimicrobial activity was obtained by functionalization by impregnation of chitosan with quaternary ammonium salts, followed by that obtained by functionalization of chitosan with phosphonium. The lowest antibacterial and antifungal effects were expressed by Ch-THIO and Ch-MBT, but new materials obtained with these extractants may be precursors with a significant role in the direct control of active molecules, such as cell growth factors or cell signaling molecules.

## 1. Introduction

Bacteria and fungi permanently adapt over the usual antibacterial and antifungal therapies, being the reason why multidrug-resistant strains and mixed infections appear. This behavior is a major problem concerning all life aspects and quality of life; its major impact is seen in medicine [1,2,3], but also in the food industry [4,5]. This tendency is a worrying one, which is why research is focused on developing new molecules that are potentially active against these microorganisms and that could be widely used with outstanding antibacterial or antifungal results [6,7]. Over time, various materials with bacteriostatic [8,9,10], bactericidal [11,12,13,14,15], fungistatic [10,16] and fungicidal [11,12,17,18,19,20] properties, against a wide spectrum of microorganisms, have been studied. It is known that materials with antimicrobial activities are of interest, both for academic studies and for industry. Such features allow their usage in the food industry or in medicine. As a matter of fact, polymers and copolymers obtained by grafting with quaternary ammonium or phosphonium salts have special properties such as biocompatibility, biodegradability, or increased antimicrobial activity [3,21,22].

Because most antibacterial materials are synthetic polymers [23], their biocompatibility and biodegradability are much more limited than those of natural polymers, such as cellulose, chitin, chitosan and their derivatives [3,12,15,24,25,26,27,28]. Although chitosan is naturally abundant [8], it is limited in terms of its reactivity and processability, which is why it is functionalized with various active groups, so as to improve its properties [12,21,25,29,30,31,32,33,34]. On the other hand, more and more pathogens have developed antibiotic resistance over time, which has led to a new approach for the development of materials with antimicrobial properties, from the category of biocompatible ones, such as chitosan [35,36].

Chitosan is a cheap and non-toxic, environmentally friendly material, which has the advantage that in the basic structure there are active amino and hydroxyl groups. These groups are easy to be functionalized, resulting in new materials, which often have increased antimicrobial activities with non-functionalized support material. Although both native chitosan and its derivatives are effective as antimicrobial agents, there is a clear difference between them, determined inter alia by the type of reactive group attached [37,38,39].

Chitosan has been shown to have good antimicrobial activity against a wide variety of microorganisms, including fungi [4,23,27,28,29], algae and bacteria [7,12,15,29,30,40,41,42]. However, antimicrobial action is influenced by numerous intrinsic and extrinsic factors, such as: nature of chitosan [30,34], degree of polymerization [35], molecular weight [12,28,36,37], pH [31,36,37], temperature, targeted microorganism [4,9,27,29,31,35,38], substrate chemical composition [40,43,44,45].

The interest in obtaining biocompatible materials, with bacteriostatic or bactericidal activity, is determined by the fact that microbial infection remains a challenge in many areas of modern life, such as new medical-surgical procedures [40,41,42], water treatment systems [43,44,45], the textile industry [46], the production of food packaging [37], but also in the control of food storage conditions [4,5,13]. The aim of this study is to obtain new materials by modifying the matrix of support biopolymers with intrinsic antimicrobial properties, chitosan (Ch), in order to improve antibacterial and antifungal activity, using a simple technique of functionalization, by impregnation [46], with active groups (nitrogen, phosphorus, sulfur), without the need to meet special reaction conditions, which sometimes result in by-products. As materials with active groups were considered: dodecyl-trimethyl-ammonium bromide (DDTMABr), tetradecyl-trimethyl-ammonium bromide (TDTMABr), hexadecyl-trimethyl ammonium chloride (HDTMACl), dodecyl-triphenyl bromide (DDTPPBr), tri n-butyl-hexadecyl phosphonium bromide (HDTBPBr), 2-mercaptobenzothiazole (MBT), thiourea (THIO).

## 2. Materials and Methods

### 2.1. Materials: Obtaining and Characterization

To obtain the materials, chitosan (40 mesh, 90% degree of deacetylation and molecular weight of 1–3 × 10^5^, Acros Organics, Waltham, MA, USA) was used as a support, and dodecyl-trimethyl-ammonium bromide (DDTMABr—Acros Organics, Waltham, MA, USA), tetradecyl-trimethyl-ammonium bromide (TDTMABr—ThermoFisher, Waltham, MA, USA, purity 98%), hexadecyl-trimethyl ammonium chloride (HDTMACl—ThermoFisher, Germany, purity 98%), dodecyl-triphenyl phosphonium bromide (DDTPPBr—ThermoFisher, Germany purity 98%), tri n-butyl-hexadecyl phosphonium bromide (HDTBPBr—ThermoFisher, Germany, purity 98%), 2-mercaptobenzothiazole (MBT—Janssen Chemistry, Bucharest, Romania), thiourea (THIO—Fluka AG, Charlotte, NC, USA), known for the pending groups present in structure (N, P, S). Weighed 0.1 g of the extractant, which was dissolved in 25 mL of distilled water. It was put in contact with 1 g of chitosan, respecting the support:extractant ratio = 10:1 (*w*/*w*). The contact time for functionalization was 24 h, then dried in an oven (POL-EKO SLW 53 STD, Wodzisław Śląski, Poland) for 24 h at 323 K. The obtained material was characterized by scanning electron microscopy, SEM and X-ray energy dispersion (EDX), using the X-ray energy dispersion spectrometer, FEI Quanta FEG 250 instrument (FEI, Hillsboro, OR, USA).

### 2.2. Preparation of Bacterial Cultures

The presence of aerobic and facultatively anaerobic heterotrophic bacteria was highlighted by inoculation in Petri dishes by the process of incorporation into a non-selective solid nutrient medium. For all seedings, a completely dehydrated culture medium was used, and the manufacturer’s instructions were followed (Plate Count Agar—produced by Merck (Peptone from Casein 5.0 g/L; Yeast Extract 2.5 g/L; D (+)—Glucose 1.0 g/L). L; Agar 14.0 g/L; Final pH: 7.2 ± 0.1 at 303 K). The bactericidal effect was highlighted by determining the number of colony forming units (CFU/mL) cultured at 310 K.

The bacterial inoculum was deposited on the liquid culture medium, then embedded. Each time, 1 mL of bacterial inoculum (approx. 1 × 10^8^ CFU/mL) was taken and was poured onto the molten culture medium and cooled to 318 K. The Petri dishes were incubated for 48 h in a standard thermostat at 310 K.

For each set of experiments (3 repetitions), a Petri dish was seeded containing the control sample (M0—control sample), consisting of culture medium and 1 mL of bacterial inoculum, without support material (Ch) and without functionalized material. In addition, for each set of experiments (3 repetitions), a Petri dish containing culture medium, bacterial inoculum and non-functionalized support material (M1) was seeded. 

For each functionalized support material, various ratios were established between the support material and the extractor with which the material was functionalized. Subsequently, these ratios constituted the solid material that was deposited in the Petri dish. Ratios support material:functionalizing material (extractant) were: 1:0.003; 1:0.006; 1:0.009; 1:0.012; 1:0.025; 1:0.050; 1:0.075; 1:0.1; 1:0.2; 1:0.3; 1:0.4; 1:0.5.

An amount of non-functionalized or functionalized solid material of about 0.2 g was used in each Petri dish, which was distributed as evenly as possible in the culture medium and 1 mL of the bacterial inoculum (approx. 1.5 × 10^8^ CFU/mL), at an amount of culture medium of about 15 mL. Bacterial colony counting was performed automatically using the automatic bacterial counter, Flash & Go (IUL Instruments SA, Barcelona, Spain), produced by IUL. 

Subsequently, to establish the effectiveness of the functionalized material, microbiological control tests were performed on 1 × 10^5^ CFU/mL from reference microbial strains (Thermo Scientific, Waltham, MA, USA), including two Gram-negative bacteria (*Esherichia coli* ATCC 25922, *Pseudomonas aeruginosa* ATCC 27853), a Gram-positive bacterium (*Staphylococcus aureus* ATCC 25923) and a fungal species (*Candida parapsilosis* ATCC 22019). Ethanol was used as a negative control.

The efficiency of the functionalized material on the reference strains was expressed as the rate of inhibition of bacterial growth [35], calculated as the ratio between the number of colony-forming units on the functionalized and non-functionalized material, expressed as a percentage ratio, according to the equation:

(1) $$inhibition\;rate=\frac{\left[UFC_{control}-UFC_{test}\right]}{UFC_{control}}\times 100$$

*UFC_control_* = the number of colonies on the control plate.

*UFC_test_* = the number of colonies on the test plate.

Through this procedure, the optimal working ratio was established (support:extractant), representing the ratio at which no bacterial growth was observed or the rate of inhibition of bacterial growth was the highest. Total bactericidal effect was considered where the rate of inhibition of bacterial growth was 100%.

## 3. Results and Discussions

### 3.1. Characterization of Materials Obtained by Functionalization of Chitosan

The materials obtained by functionalization, by impregnation were characterized by scanning electron microscopy, SEM, X-ray energy dispersion, EDX and infrared spectroscopy with Fourier transform, FT-IR to highlight the presence of the extractant (DDTMABr, TDTMABr, HDTMACl, DDTPPBr, HDTBPBr, MBT and THIO) on the support surface (Ch).

**Figure 1:**
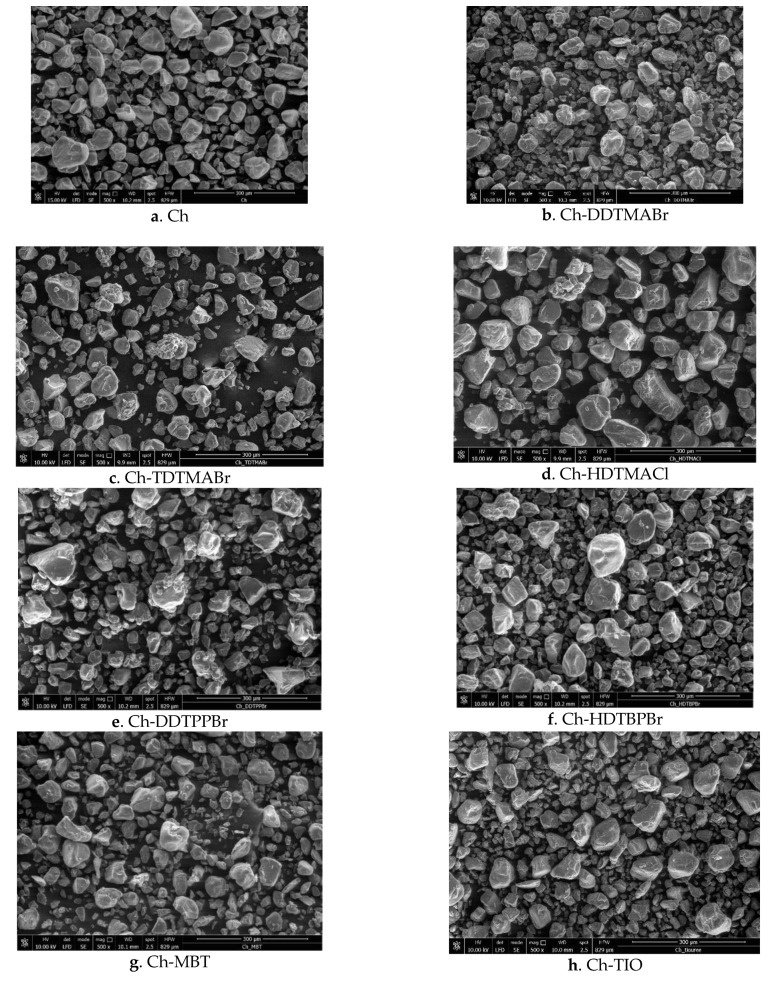
(**a**–**h**) SEM images for materials obtained by functionalizing chitosan with different extractants.

The SEM images (Figure 1) show the morphology of the support material, (Ch), but also of the materials obtained after the functionalization of chitosan with the seven extractants (DDTMABr, TDTMABr, HDTMACl, DDTPPBr, HDTBPBr, MBT and THIO). In Figure 1b,c,f, the presence of a white compact layer on the chitosan granules can be observed. In the case of the remaining samples, we can observe the presence of some white dots associated with the presence of used extractants. From these images, you can observe a slight modification in the morphology of materials obtained after chitosan functionalization.

**Figure 2:**
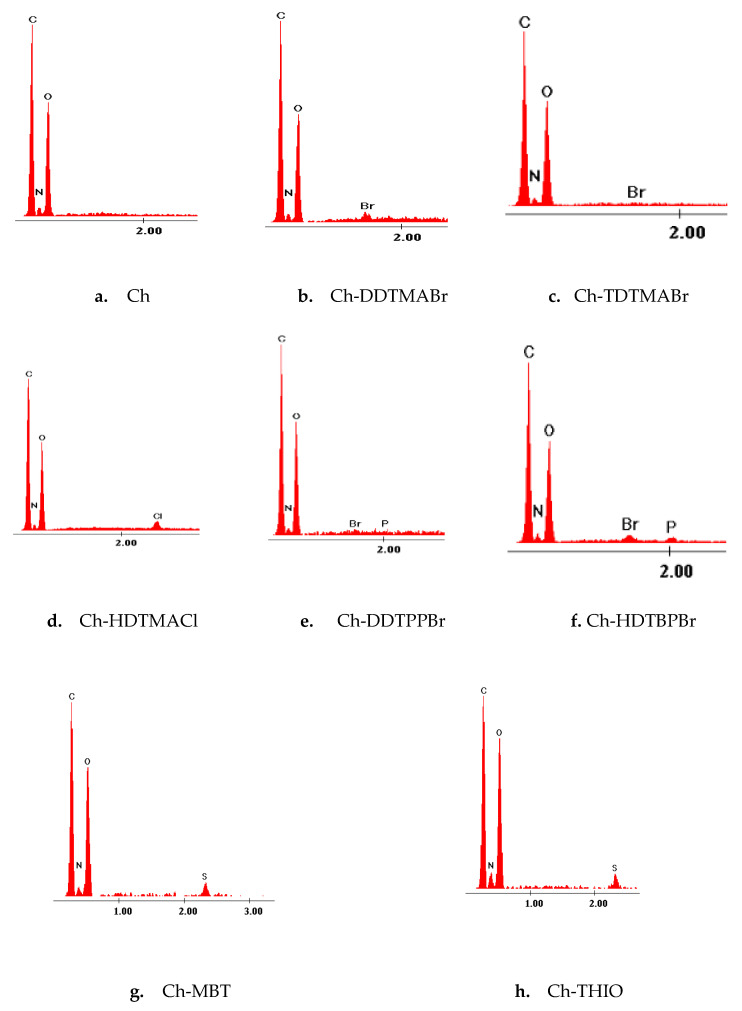
(**a**–**h**) EDX spectra for materials obtained by functionalizing chitosan with different extractants at ratio 10:1.

From the X-ray energy dispersion spectra, EDX (Figure 2), the presence of chitosan-specific C, N and O is observed. In addition, the presence of N, P or S elements specific to the existing groups in the structure of the extractants is observed.

By these means:

1. the presence of DDTPPBr and HDTBPBr on the surface Ch is highlighted by the appearance of the specific P and Br pick;
2. the presence of TDTMABr, HDTMAC1 and DDTMABr on the surface Ch is highlighted by the appearance of the specific pick N and Br or N and Cl depending on the compound;
3. the presence of MBT and THIO on the surface Ch is highlighted by the appearance of the specific pick N and S.

These picks are small in size because the amount of extractant that is functionalized is relatively small, the ratio of chitosan:extractant is 10:1.

**Figure 3:**
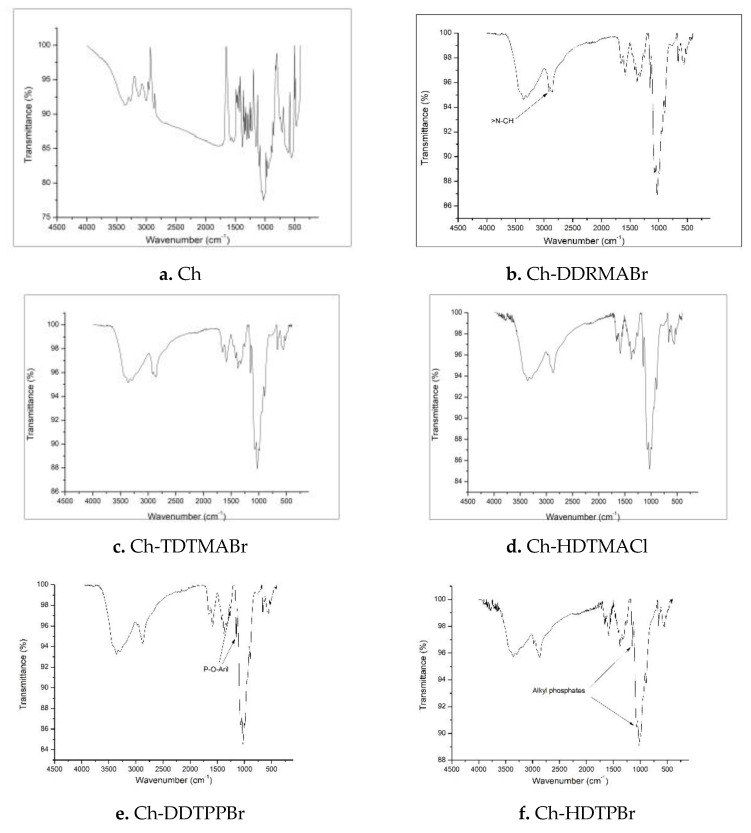

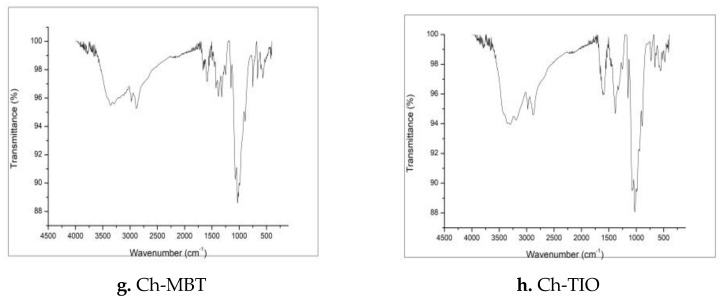
(**a**–**h**) FT-IR spectra for materials obtained by functionalizing chitosan with different extractants.

The main objective of obtaining infrared spectra with the Fourier transform (Figure 3) is to determine the functional groups of the obtained materials. These functional groups adsorb IR radiation at characteristic frequencies, which is why these spectra highlight specific functional groups. Table 1 shows the groups specific to chitosan and those specific to extractants.

### 3.2. Studies on the Antimicrobial Activity of Materials

#### 3.2.1. Chitosan Functionalized with Quaternary Nitrogen Salts

(a). Studies to Establish the Optimal Ratio of Chitosan:Extractant

In order to establish whether Ch or functionalized Ch have bactericidal properties, we performed microbiological analyses to highlight the behavior of these materials in the presence of a consortium of heterotrophic, Gram-positive and Gram-negative bacteria. The use of a complex bacterial inoculum, with heterotrophic bacteria, was preferred for material testing because the study of the behavior of a bacterial consortium from the natural environment is preferable in the case of the preliminary study on a newly synthesized material, compared to the effect obtained on a single bacterial species.

To establish the lowest ratio between Ch and the utilized extractant, which has a bactericidal effect on the bacterial inoculum, each material was inoculated into culture medium and incubated for 48 h at 310 K. The total number of colonies was determined, reported to the control sample M1 (non-functionalized Ch), respective to the control sample M0 (bacterial inoculum and culture medium). This ratio, in which the complete absence of microbial growth was observed or the calculated inhibition rate had the highest value, was considered the optimal, minimum necessary ratio, which produces total bactericidal effect or shows significant bactericidal effect. 

**Figure 4:**
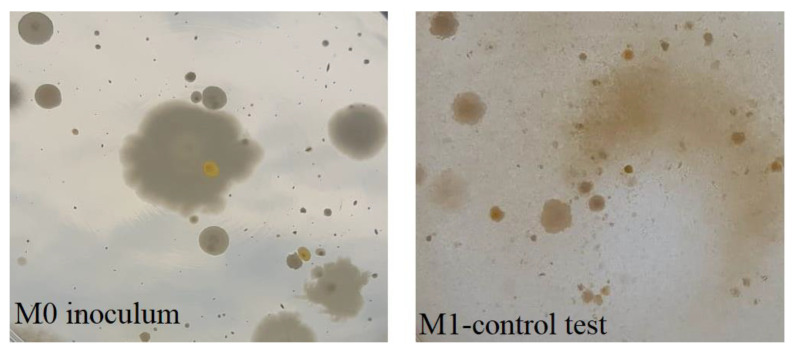
Bacterial inoculum culture (**M0**) and Non-functionalized chitosan control test culture (**M1**) (magnification 1×).

Chitosan itself has bactericidal activity (Figure 4). This was demonstrated by inoculating an M1 control sample (where non-functionalized support material, chitosan, was added), which was compared with the M0 control sample (where only the bacterial inoculum and culture medium were inoculated).

Comparing the two samples, M0 and M1, it can be seen that the support material, chitosan itself, has bactericidal properties, as the growth of bacteria on the surface of the culture medium was inhibited, with an inhibition rate of about 41.3% (from 1.45 × 10^8^ col/mL to 8.5 × 10^7^ col/mL). It should not be neglected that in the present study, chitosan with medium molecular weight (1–3 kDa) [47] and high degree of deacetylation (90%) was used, whose intrinsic properties are favorable for the manifestation of bactericidal effect [43,48]. 

**Figure 5:**
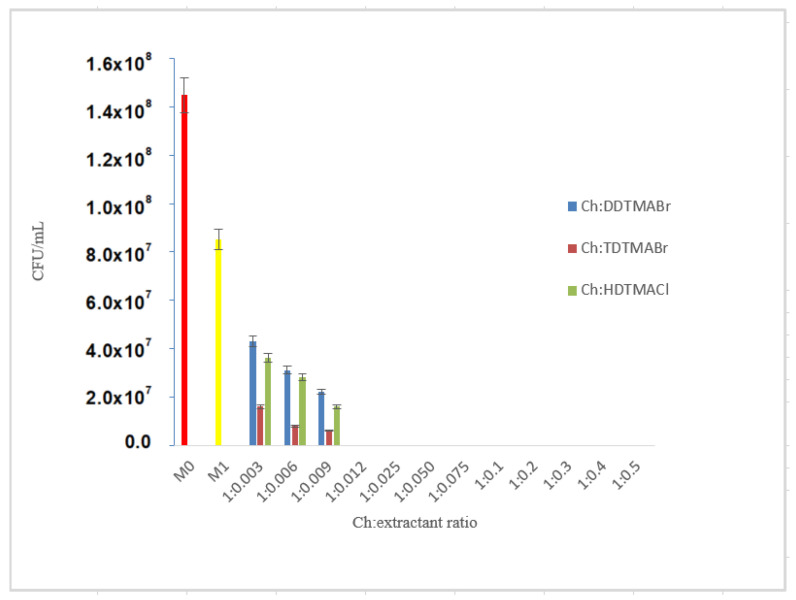
Comparison of control sample (M0)—Ch (M1)—Ch:functionalized with quaternary ammonium salts.

Figure 5 shows result for after examination of the series of bacterial cultures where the quaternary ammonium salts was used for functionalizing. In the case of DDTMABr extractant, it was found that at support:extractant ratios greater than 1:0.009 no bacterial growth took place. Consequently, the optimal functionalization ratio Ch:DDTMABr that produces total bactericidal effect is 1:0.012. Regarding the Ch-TDTMABr material, the analysis of the Petri dishes led to the same conclusions: by functionalization, its bactericidal capacity is increased, the Ch-TDTMABr material at functional ratios higher than 1:0.009 shows the total bactericidal effect. Consequently, the optimal functionalization ratio Ch:TDTMABr that produces total bactericidal effect is 1:0.012. After examination of a series of bacterial cultures in the case of Ch-HDTMACl material, it was found that starting with the ratio support: extractant of 1:0.012, no bacterial growth took place. Consequently, the optimal functionalization ratio Ch:HDTMACl that produces total bactericidal effect is 1:0.012.

All three extractants studied so far (DDTMABr, TDTMABr, HDTMACl) are quaternary ammonium salts with which chitosan has been functionalized, at which the length of the alkyl chain which represents the spacer chain distancing arm (dodecyl, tetradecyl, hexadecyl) differs, but which has the chain on the pendant group, N, identically (methyl). For these extractants, the bactericidal response is similar, as evidenced by the rate of total inhibition of bacterial growth, to Chitosan:extractant at identical functionalization reports.

The hydrophobicity and cationic charge of the introduced substituent strongly affect the antibacterial activity of the quaternary chitosan derivatives [29]. However, it has been shown that the length of the alkyl chain that separates the extractor chain brings hydrophobic input and contributes through the cationic charge to the manifestation of the total bactericidal effect of functionalized chitosan, regardless of the extractant—quaternary salt used. 

(b). Antibacterial and Antifungal Effect of Nitrogenous Materials on Reference Strains

To establish the effectiveness of the synthesized materials, microbiological control tests were performed on *E. coli* (ATCC 25922), *P. aeruginosa* (ATCC 27853), *S. aureus* (ATCC 25923) and *C. parapsilosis* (ATCC 22019). A 1 × 10^5^ CFU/mL volume of each microbial reference strain inoculum was seeded. The tests were performed in three repetitions, using both the optimal ratio previously established and other ratios support:extractant, in order to be able to observe the bacterial behavior at lower or higher ratios than the optimal ratio, thus confirming the minimum required ratio for total bactericidal effect.

Due to the different composition of the walls in Gram-positive and Gram-negative bacteria, the interaction of chitosan and its derivatives is different for these two types of bacteria, a fact proven in this paper and supported in specialized studies [21,22,39,49,50,51,52,53,54,55,56].

Calculating the rate of inhibition of bacterial growth for the four used strains, under the action of chitosan, compared to the control sample (ATCC inoculum), respectively for chitosan derivatives compared to chitosan, the data are as follows (Table 2):

Differences in the cell wall structure of Gram-positive and Gram-negative bacteria led to slightly lower inhibitory effects against Gram-positive bacteria compared to Gram-negative bacteria for non-functionalized chitosan.

Experimentally, it has been shown that the binding site of unmodified chitosan is on the surface of the outer membrane of Gram-negative bacteria, by binding blocking the membrane permeability, so that its barrier function is disturbed, which alters the viability of the cell [30,47,49]. 

In order to obtain chitosan derivatives with high water solubility, especially at physiological pH values [7] and an improved antibacterial activity, one of the methods of functionalization of chitosan is the use of quaternary ammonium salts. This process is preferred due to the bactericidal potential [7] of these salts.

Compared to the classical chemical methods used to prepare the quaternary salts of chitosan, in this paper new material was obtained through functionalization by impregnation. This is a very fast, cheap and easy to manage method that does not require special working conditions. Functionalization is achieved by putting in contact the support material (chitosan) and the extractant (previously dissolved in water) for a period of time, followed by drying the material obtained.

Regarding the manifestation of the antibacterial effect on *P. aeruginosa*, the antibacterial activity of the quaternary salt derivatives of chitosan decreased as the distance of the positive charge from the polymer backbone (given by the quaternary nitrogen) increased.

Table 3 shows the structures of the extractants that have a nitrogen heteroatom in their composition.

Analysis of the stable conformations of these three extracts, which are represented in Table 3, shows that the closest positive charge, given by quaternary nitrogen, to the basic structure of chitosan is at TDTMABr, followed by DDTMABr and HDTMACl. This is in line with the calculated inhibition rate for *P. aeruginosa* corresponding to the highest value Ch:TDTMABr material, 60.3%. For Ch:TDTMABr ratio = 1:0.1 the CFU/mL was 3.18 × 10^4^). Considering the order of length increase in the alkyl substituent which determines the distancing of the positive charge given by the quaternary nitrogen towards the chitosan basic bone, the other two salts are: DDTMABr and HDTMACl, with the inhibition rate calculated for the ratio Ch:extractant = 1:0.1 of 57.2% (3.43 × 10^4^ CFU/mL) and, respectively of 29.2% (5.67 × 10^4^ CFU/mL) for *P. aeruginosa*. In the case of Ch-TDTMABr, even when reducing the ratio support:extractant 10 times, the inhibition rate is kept at approximately the same order of magnitude (55.2% for the ratio 1:0.012 compared to 60.3% for the ratio 1:0.1). In the case of Ch-DDTMABr, the 10 times reduction in the Ch:extractant ratio determined the reduction in bactericidal efficiency by almost a half, the inhibition rate being reduced from 57.2% to 31.7%. In the case of Ch-HDTMACl, when the Ch:extractant ratio was 10 times reduced, the inhibition rate for *P. aeruginosa* decreased from 29.2% to 13.1%, which means a substantial reduction in bactericidal efficacy. Compared to the *E. coli* strain, although also Gram-negative bacteria, the Ch-TDTMABr material showed total bactericidal activity, with a bacterial growth inhibition rate of 100%, while the Ch-DDTMABr and Ch-HDTMACl materials have a rate of approximately equal inhibition, 54.9% and 52.7%, respectively. In the case of *E. coli,* strain was observed that the inhibition rate is inversely proportional to the distance between chitosan molecule and extractant positive charge, as it is in the case of *P. aeruginosa*.

Therefore, the antibacterial activity of the materials with the same functional group bound to the quaternary nitrogen (in this case the methyl group) depends on the positioning of the positive charge on the polymer backbone, which is in agreement with the literature data [29,39]. In conclusion, for the same polymer-linked functional group by alkyl substituents of different lengths, the antibacterial activity is higher when the positive charge (or cationic segment) is closer to the initial structure of the polymer and decreases when the functional group is present at an increasing distance from the base structure of the polymer [46]. In this case, it seems that the one that affects the interaction with the outer membrane of Gram-negative bacteria is the conformation of the polymer derivative. As the length of the alkyl substituent chain increases, the polymer will likely tend to adopt a conformation that is less favorable for efficient binding to the anionic components of the bacterial membrane and therefore the antibacterial activity is reduced. 

*P. aeruginosa* is known to be a strain resistant to most antibiotics commonly used to treat infections [60,61]. This is a cause for the lower bactericidal response of the three materials whose extractors are quaternary ammonium salts on this bacterium, compared to the bactericidal response of the same materials on the bacterium *E. coli*, also Gram-negative.

For *S. aureus*, Gram-positive bacterium, regardless of the working ratio Ch:extractant, for all three materials obtained by functionalization of chitosan with quaternary ammonium salts, the inhibition rate was 100%, the bactericidal activity being maximum. Thus, the lack of the outer membrane in the case of Gram-positive bacteria facilitates an antibacterial effect [56]. Another argument is the presence of teichoic acids, with anionic character [57], which have a high affinity for cationic derivatives, such as quaternary salts.

In the case of *S. aureus*, the interdependence between the positive charge position of quaternary nitrogen and the basic skeleton of chitosan could not be proved. The wall of Gram-positive bacteria has a thicker layer of peptidoglycan; the extractants used are based on quaternary ammonium salts of cationic character adhered much more easily to the surface of the cell wall, thus damaging its structure, which subsequently causes the cell contents to leak and implicitly the death of bacterial cells. This consideration is in line with data from the literature, which claims that the structure of the cell wall in Gram-positive bacteria is destroyed, and cell viability is disrupted by the action of hydrophobic compounds [47,50,62]. In this case, the conformation of the alkyl substituent seems to play a secondary role, because the material must not be able to penetrate through the pores of the outer membrane (as occurs in Gram-negative bacteria), but adhesion to the surface of the bacterial cell is sufficient. Thus, more important is the hydrophobic character given by the introduction of the alkyl chain from the quaternary salt. This is based on data from the literature: the site of action of antibacterial agents is the cytoplasmic membrane of bacteria [61]; the main constituents of the cytoplasmic membrane are membrane proteins and phospholipids. Thus, the materials obtained by functionalizing chitosan with quaternary ammonium salts, which have a long alkyl chain structure, interact strongly with cytoplasmic membranes due to a hydrophobic affinity between the introduced alkyl chain and phospholipids, leading to a higher antibacterial activity [53]. Kong et al. showed the importance of the equilibrium between the hydrophilic character of the bacterial cell and the hydrophobic character of the tested material. They point out that the potential for interaction between chitosan derivatives (which have quaternary ammonium salts in the structure) and the bacterial cell wall is strongly influenced by this balance [53]. 

In Gram-negative bacteria, interactions with the cytoplasmic membrane are conditioned by the penetration of the material through the porins in the outer membrane, which causes the antibacterial effect to be slowed down compared to that expressed upon Gram-positive bacteria [55,56,63].

Regarding the antifungal activity of chitosan and the materials obtained by its functionalization with quaternary ammonium salts, they showed special antifungal activity on *C. parapsilosis*. Research in the field has shown that the antifungal activity of chitosan is correlated with a higher amount of unsaturated fatty acids present in the cell membrane of some species of fungi [64]. The content of unsaturated fatty acids positively influences the fluidity of the membrane, which further influences the susceptibility to the action of chitosan and the materials obtained by its functionalization [36]. This is because greater membrane fluidity tends to lead to a negative charge on the cell membrane [23,64], thus facilitating the binding of cationic chitosan to the fungal cell membrane, leading to fungal cell death [65]. 

On the other hand, in the fungal cell wall in some species, including *C. parapsilosis*, manoproteins are present, which have the role of cell wall integrity, adhesion to host tissues, virulence and in establishing a protective immune response of host cells [66]. These manoproteins contain phosphate groups that also influence the negative charge of the fungal cell wall [36,67]. The decisive role of the antifungal properties of the materials synthesized in this study can be attributed to their hydrophobic nature, which increases the interaction with the fungal cell wall, as has been pointed out in a number of important studies [12,19,67].

Therefore, regarding the effect of the materials obtained by the functionalization of chitosan with quaternary ammonium salts on the antifungal activity, the contribution of the hydrophobic character is obvious, reflected in the inhibition rate, regardless of the length of the carbon atom chain. The increased antifungal activity of the material can be explained by the electrostatic interaction between the fungal cell and the permanent positive charge due to quaternization. The explanation is that the more positively charged the studied materials (they have a more pronounced cationic character), the more they can interact with negatively charged microbial components, which leads to antifungal activity [20]. Thus, the inhibition rate for *C. parapsilosis* is maximum, except for the ratio 1:0.012 when Ch-DDTMABr material was studied. It is possible that in this case, the extractant, DDTMABr, has a shorter carbon atom chain than the other extractants. We can appreciate that there is a minimum threshold necessary to be reached in terms of hydrophobic intake, so that fungicidal activity can be expressed. This is in line with the observation made by Kong et al. [54] in bacterial studies, where the equilibrium between the hydrophilic character of the bacterial cell and the hydrophobic character of the tested material plays an important role. It has been shown that, in order to exert the total antifungal activity for a synthesized material (RI = 100%), it is necessary to achieve a balance between the hydrophobic character of the tested material and the hydrophilic character of the fungal cell.

#### 3.2.2. Chitosan Functionalized with Phosphorus Heteroatom

(a). Studies to Establish the Optimal Ratio Support:Extractant

**Figure 6:**
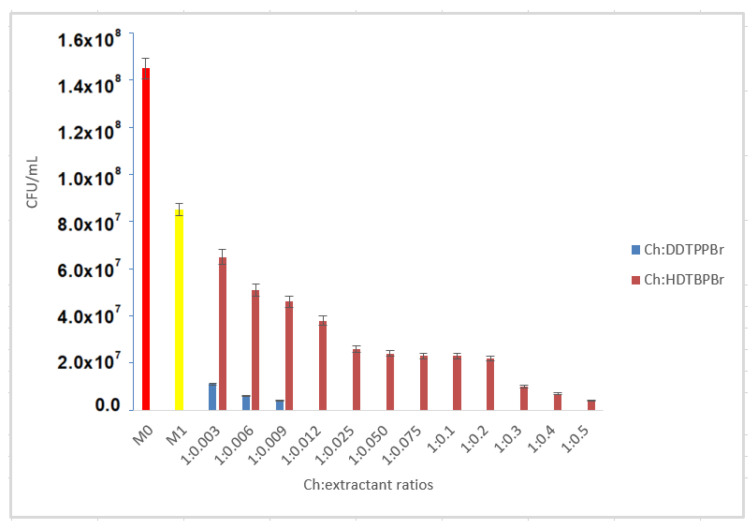
Comparison of control sample (M0)—Ch (M1)—Ch:functionalized with phosphorus heteroatom.

Analyzing the M1 control sample, it was observed that only chitosan (non-functionalized) allows the development of bacteria on the surface of the culture medium. So, it can be concluded that by functionalizing chitosan with DDTPPBr, regardless of functionalization ratios, bactericidal capacity is positively influenced (Figure 6). Studying a series of bacterial cultures in which the DDTPPBr extractant was used, it was found that at support: extractant ratios higher than 1:0.009 no bacterial growth took place. Consequently, the Ch-DDTPPBr material had a total bactericidal effect starting with the ratio Ch:DDTPPBr = 1:0.012.

When chitosan was functionalized with HDTBPBr, the bactericidal capacity was modified, being directly proportional to the increase in the amount of extractant added. It is therefore observed that by adding the extractant, the antibacterial capacity is improved (Figure 6). At a Ch-HDTBPBr ratio of 1:0.012, the bacterial growth inhibition rate was 55.3%, while for the Ch-HDTBPBr ratio of 1:0.5, the bacterial growth inhibition rate was 95.3%. As no total bactericidal effect was obtained in any of the working ratios, the optimal ratio Ch:HDTBPBr was considered 1:0.5.

(b). Antibacterial and Antifungal Effect of Phosphorus Materials on Reference Strains

Inhibition of *S. aureus* strain growth was also obtained by using the materials Ch-DDTPPBr and Ch-HDTBPBr, whose chemical structure differs both in the length of the alkyl substituent chain from the basic structure of chitosan (dodecyl and hexadecyl) to the positive charge of the active group, phosphonium, as well as by the different quaternization substituent (triphenyl and tributyl, respectively).

Table 4 shows the structures of the extractants that have a phosphorus heteroatom in their composition.

Considering the two chemical structures, DDTPPBr and HDTBPBr extractants, bactericidal activity is expected to be better in the case of DDTPPBr. This is confirmed in the case of tests on Gram-negative bacteria, where for both *E. coli* and *P. aeruginosa*, at the same ratio support:extractant, regardless of the choice of this ratio, the inhibition rate is higher for Ch-DDTPPBr, according to the data in Table 5.

The effect of Ch-DDTPPBr determines an inhibition rate of 100% on *E. coli* and 46.6% (4.28 × 10^4^ CFU/mL) on *P. aeruginosa*, for a ratio support:extractant of 1:0.1. For the same ratio support:extractant of 1:0.1, in the case of Ch-HDTBPBr on *P. aeruginosa* the inhibition rate is 28.2% (5.75 × 10^4^ CFU/mL), and by increasing the ratio Ch:extractant five times, the inhibition rate becomes similar (48.6%) to that for Ch-DDTPPBr. To establish the optimal Ch:extractant ratio, economic considerations were made.

In the case of the use for impregnation of extractants containing phosphorus heteroatom, the role in expressing their antibacterial effect can be attributed both to the hydrophobic chain of the two derivatives and to the highly hydrophobic aromatic groups in the phenyl substituent, Refs. [68,69] compared to butyl radicals, grafted on the active group, phosphonium.

Of course, if the newly synthesized material is used in the medical field, it requires new, more laborious research, but the very good results obtained on *S. aureus,* frequently encountered in nosocomial wound infections [21], entitle us to consider that newly synthesized materials have excellent antibacterial activity.

Based on the performed experiments, using the two materials, Ch-DDTPPBr and Ch-HDTBPBr, the results obtained showed total bactericidal activity on *S. aureus.* The hydrophobic influence is decisive, as both the aromatic groups and the chain of the alkyl substituent that distances the phosphorus heteroatom from the basic structure of chitosan, contributing to the increase in the hydrophobic character of the two materials. This causes strong binding to the bacterial cell surface, leading to a massive loss of intracellular components, leading to nutrient depletion and subsequent death of the bacterial cell [14,21,22,39,51].

Due to the potential to use chitosan derivatives with phosphorus or phosphate compounds, in the medical field [70,71], in orthopedic or dental bone implant technology [72], knowledge of data on antibacterial activity of materials obtained by functionalization by impregnation of chitosan with phosphorus-containing extractants is essential. The fact that these materials containing chitosan functionalized with groups grafted on the phosphorus atom have biomedical potential, determines a real concern for the improvement of their properties, correlated with the increase in the bone and tissue regeneration capacity [25,70,71,72,73]. 

In terms of antifungal effect of these materials obtained by functionalizing chitosan with extractants containing phosphorus heteroatom, the hydrophobic influence is decisive, as was also observed in the case of nitrogen-based materials. Both the aromatic groups (phenyl) and the length of the alkyl substituent chain that distances the phosphorus heteroatom from the basic structure of the polymer, contribute to increasing the hydrophobic character of the two materials, which determines their strong binding to the fungal cell surface. Consequently, the rate of inhibition of *C. parapsilosis* growth is 100%.

#### 3.2.3. Chitosan Functionalized with Sulfur Compounds

(a). Studies to Establish the Optimal Ratio Support:Extractant

**Figure 7:**
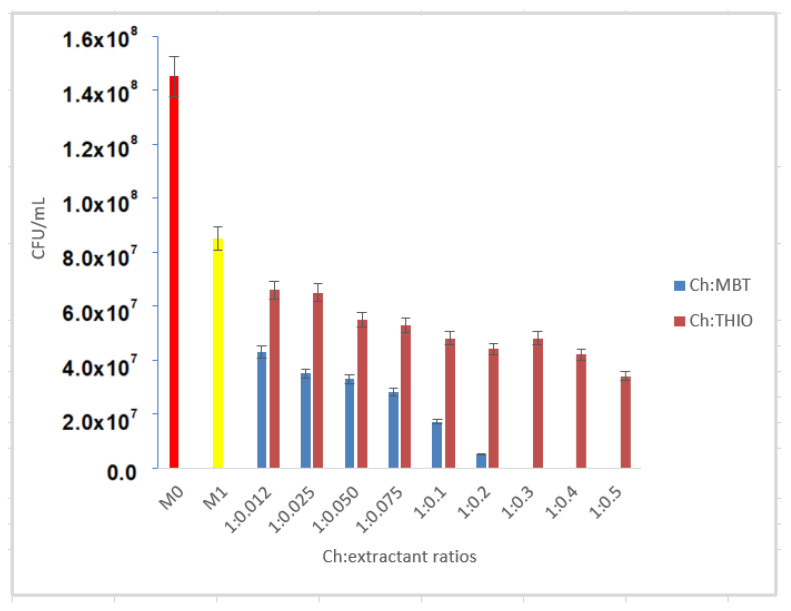
Comparison of control sample (M0)—Ch (M1)—Ch: functionalized with sulfur compound.

Compared to the analysis of the M1 control sample, when non-functionalized chitosan allowed the development of bacteria on the surface of the culture medium, it can be concluded that by functionalizing chitosan with MBT, with an increasing functionalization ratio, bactericidal capacity is positively influenced (Figure 7). After examination of the series of bacterial cultures in which MBT was used as an extractant, it was found that at the ratio Ch:MBT = 1:0.3 there was no bacterial growth. Consequently, for the Ch-MBT material, the ratio that showed total bactericidal effect and is considered optimal working ratio is 1:0.3.

Studying the series of bacterial cultures in which thiourea was used as an extractant, it was found that for no Ch:THIO ratio there was no total bactericidal effect.

Compared to the analysis of the M1 control sample, it can be concluded that by functionalizing chitosan with THIO, with the increase in the functionalization ratio, the bactericidal capacity is also positively influenced (Figure 7). For the functionalization ratio Ch:THIO = 1:0.012, a bacterial growth inhibition rate of 22.4% was obtained, while at the maximum working ratio Ch:THIO = 1:0.5 the calculated inhibition rate was 60%.

To establish the optimal working ratio, given that we cannot talk about the total bactericidal effect, we considered the optimal ratio Ch:THIO = 1:0.5.

(b). Antibacterial Effect of Sulfur Materials on Reference Strains

Although the functionalization of chitosan to obtain bio-functional materials with polycationic character is very widespread among research in recent years, of great interest is the strategy to reduce the positive charge of protonated amino groups presented on the polymeric surface of chitosan, so as to obtain anionic derivatives. Anionic chitosan derivatives have good applicability in the medical field, in the case of tissue regeneration and have selective antimicrobial activities. Sulfonate derivatives of chitosan are anionic in character and have a heparin-like structure, being capable of electrostatic interaction with positively charged cell growth factors, stabilizing them and preventing their degradation [74].

In this paper, we wanted to highlight the existence of antibacterial or antifungal activity of chitosan derivatives that are not anionic, as the functionalization is not done with sulfonic groups, but with extractants that contain sulfur in the form of the -thiol group. Compounds containing the thiol group in ionized form have high reactivity and have many biological functions: the role of metal cofactors, participants in the structure of proteins, precursors of iron-sulfur groups involved in the electron transport chain that provide energy to organisms [75,76].

Table 6 shows the structures of the extractants that have sulfur atoms in their composition.

Due to the recommendations of MBT as an antibacterial and antifungal agent [79,80], we resorted to testing the material obtained by functionalizing through impregnation of chitosan with MBT as a potential antibacterial and antifungal agent.

The total antifungal effect of the Ch-MBT material on *C. parapsilosis* was observed for the ratio Ch:MBT = 1:0.3, at which the inhibition rate was 100%. For lower functional ratios, the calculated inhibition rate is shown in Table 7.

According to the literature, we can say that thiol groups are necessary in order to obtain the antifungal and antibacterial effect [79,80,81]. Regarding the antibacterial activity of the Ch-MBT material, in most studies, the effect of the extractant MBT is considered to be more bacteriostatic than bactericidal [20,81,82]. In our work, the antibacterial activity of the Ch-MBT material, by increasing the Ch:extractant ratio, the MBT concentration increases and implicitly it is observed to obtain a maximum rate of bacterial inhibition in the case of *S. aureus* and *C. parapsilosis*. Hence the conclusion that the bactericidal or fungicidal mode of action of the Ch-MBT material is similar, in the case of Gram-positive bacteria and that of fungi, at least for fungi with a cell wall similar to *C. parapsilosis*.

Regarding the effect of Ch-MBT material on Gram-negative bacteria, the bactericidal effect is much less expressed, as evidenced by the maximum inhibition rate obtained of 38.8% (4.9 × 10^4^ CFU/mL) for *P. aeruginosa* and 72.3% (2.08 × 10^4^ CFU/mL) for *E. coli*, respectively, at the maximum studied ratio, Ch:MBT = 1:0.5. If we compare the efficiency of the Ch-MBT material at a ratio Ch:MBT = 1:0.012, the inhibition rate for *P. aeruginosa* it is 22.8% (6.18 × 10^4^ CFU/mL), and for *E. coli* the inhibition rate is 25.8% (5.57 × 10^4^ CFU/mL), without significant differences. 

As long as the replacement of hydrogen in position 4 of the phenyl ring in position 6 of the benzothiazole ring with an electron-attracting nitro group leads to complete loss of activity [81], it is expected that by binding the MBT extractant to the NH_2_ group of chitosan the antibacterial activity is substantially reduced, compared to materials that have a polycationic character (materials obtained by functionalization with nitrogen or phosphorus extractors). However, by binding the ionized thiol group to the nitrogen in the structure of the chitosan, a derivative (R-S-N) similar to nitrosothiols is formed, which has a very important role in the cellular redox cycle or in cellular signaling processes [76,83].

The functionalization of the chitosan biopolymer with MBT determined the improvement of the antimicrobial activity of native chitosan, proven by obtaining a higher inhibition rate in the case of CH-MBT material, compared to native chitosan, even for the minimum functionalization ratio Ch:MBT = 1:0.012, as shown in Table 7. Under these conditions, in the case of Ch:MBT materials, the ratio support:extractant which showed selective bactericidal activity could be used in the medical field or in the agricultural industry.

Thiourea, having the ability to donate electrons through oxygen, nitrogen and sulfur in its structure, offers a multitude of binding possibilities [84]. Materials obtained by functionalizing chitosan with thiourea [82] are known due to the role of ligands, able to coordinate a series of metal ions, but also as neutral ligands, monoanions or dianions [85]. According to studies, the efficacy of the chitosan derivative obtained by functionalizing Ch with thiourea against Gram-positive bacteria is higher than against Gram-negative bacteria [84]. The literature data and data obtained in the present study are in agreement, regardless of the studied Ch:thiourea ratio. The inhibition rate for *S. aureus* was higher than that corresponding to *P. aeruginosa* and *E. coli* (see Table 7). 

Regarding the antifungal effect of Ch-THIO derivatives, the literature emphasizes the predominant role of concentration, effect of molecular weight and degree of deacetylation of chitosan [43,86]. These arguments underlie the conclusion that the antifungal mechanism of action of chitosan and its derivatives is based on the electrostatic interactions of the positive charges of the polymer with negatively charged phospholipids in cell membranes [20,67]. However, due to the dynamics of the composition of the fungal cell wall [2,23,63], a clear mechanism for the antifungal effect is difficult to establish. It can be concluded that the antifungal effect of chitosan and its derivatives is dependent on the type of fungus on which it acts [12,67].

The functionalization of chitosan with sulfur-based extractants, compared to the functionalization of the same support with nitrogen and phosphorus-based extractants, showed a very low bactericidal and fungicidal effect in the case of sulfur derivatives. However, sulfur derivatives open up the opportunity for “nitro-thiol” compounds whose role could be particularly important for the creation of biocompatible cell signaling molecules.

## 4. Conclusions

Biopolymers and their derivatives obtained by simple functionalization techniques, such as chitosan and materials obtained in this study by functionalization, by impregnation, have multiple applications: semipermeable membranes used in biotechnology, medicine, food industry, environmental protection. 

The impregnation technique used in this paper for the functionalization of chitosan can be a cheap and fast alternative for obtaining chitosan derivatives, without the need for special reaction conditions and without involving reaction by-products. On the other hand, in the case of all chitosan derivatives obtained by impregnation, increased antimicrobial properties were obtained compared to native chitosan, which leads to the conclusion that this impregnation technique can be used successfully to obtain new materials, with very good antimicrobial effect.

Regarding the obtaining of functionalized materials that have a bactericidal or fungicidal effect, the optimal result is based on the in time initiation of new materials, with adequate properties, given the permanent adaptation of bacteria and fungi to antibiotics in use.

Chitosan derivatives obtained by functionalization with quaternary ammonium salts showed very good antimicrobial activity for all microorganisms examined (except *P. aeruginosa*, known for its resistance to most common antibiotics). In addition, all materials derived from Ch functionalization with studied quaternary salts showed a higher antibacterial activity, in general, against *S. aureus* than on *E. coli*. 

Given the potential of the derivatives obtained by functionalizing chitosan with phosphorus heteroatom to be used in obtaining biocompatible materials for various medical technologies, it is certain that the use of newly synthesized materials, Ch-DDTPPBr and Ch-HDTPPBr, with very good results on *S. aureus*, is a desideratum in obtaining biomaterials with biomedical applications. In the case of derivatives obtained by functionalization, by impregnating chitosan with sulfur-containing extractants, the antibacterial and antifungal activity on *S. aureus* and *C. parapsilosis* is less obvious in the case of small functionalization ratios and increases with increasing support: extractant ratios. 

In the tested Gram-negative bacteria, the Ch:THIO effect is rather bacteriostatic than bactericidal, because of the inhibition rate of approximately the same order of magnitude, at a 50 times change of the support:extractant functionalization ratio.

Hydrophobicity correlates well with the bioactivity of substances. The different hydrophobic behavior of chitosan-derived compounds plays an important role in their mechanisms of biological activity, whether it is antibacterial or antifungal activity. Thus, chitosan can play a role in the directed control of active molecules, such as cell growth factors or cell signaling molecules.

## Figures and Tables

**Table 1:** Specific IR bands specific to the groups in Ch and extractants.

Group	IR Band (cm^−1^)
Ch	
CH_2_–OH N–H C=O C–H O–H	1380–1420 1570 1660 2870; 2924 3430
Ch-DDTMABr	
>N–CH_2_	2700–2800 (specific non-participant e^−^ in N)
Ch-TDTMABr	
>N–CH_2_	2700–2800 (specific non-participant e^−^ in N)
Ch-HDTMACl	
>N–CH_2_	2700–2800 (specific non-participant e^−^ in N)
Ch-DDTPPBr	
P–O–Aril C–O (fenil) O–H	1190–1240 1200 3500–3200
Ch-HDTBPBr	
Alkyl phosphates	1150–1180; 1080
Ch-MBT	
Aromatic ring—torsion S–C–S C–N stretching C–H; N–H	568–600 1030–1074 1250–1320 750
Ch-THIO	
N–H C=S	1520 1074

**Table 2:** Inhibition rate of bacterial and fungal growth when using nitrogen heteroatom materials.

Material	Ratio Ch:Extractant	Inhibition Rate (%)				OBS
		*S. aureus* ATCC 25923	*P. aeruginosa* ATCC 27853	*E. coli* ATCC 25922	*C. parapsilosis* ATCC 22019	
Chitosan	-	13.1	18.5	24.5	17.7	Slightly better bactericidal effect on Gram-negative bacteria
Ch:DDTMABr	1:0.012	100.0	31.7	25.8	76.0	Maximum bactericidal effect on Gram-positive bacteria
	1:0.1	100.0	57.2	100.0	100.0	
Ch:TDTMABr	1:0.012	100.0	55.2	100.0	100.0	
	1:0.1	100.0	60.3	100.0	100.0	
Ch:HDTMACl	1:0.012	100.0	13.1	52.7	100.0	
	1:0.1	100.0	29.2	69.0	100.0	

**Table 3:** Structure of extractants with nitrogen heteroatom.

DDTMABr C_15_H_34_BrN	TDTMABr C_17_H_38_BrN	HDTMACl C_19_H_42_ClN
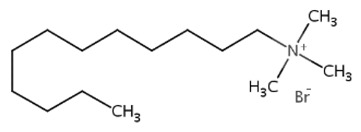 [57]	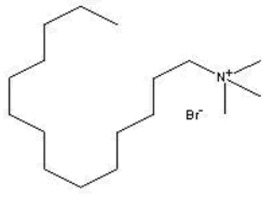 [58]	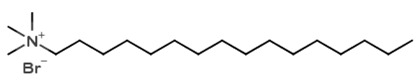 [59]

**Table 4:** Structure of phosphorus heteroatom extractants.

DDTPPBr C_30_H_40_BrP	HDTBPBr C_28_H_60_BrP
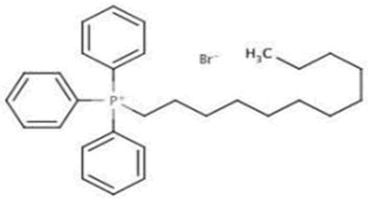 [68]	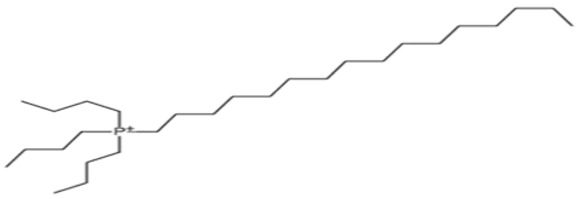 [69]

**Table 5:** Inhibition rate of bacterial and fungal growth when using phosphorus heteroatom materials.

Material	Ratio Ch:Extractant	Inhibition Rate (%)				OBS.
		*S. aureus* ATCC 25923	*P. aeruginosa* ATCC 27853	*E. coli* ATCC 25922	*C.parapsilosis* ATCC 22019	
Ch:DDTPPBr	1:0.012	100.0	31.0	100.0	100.0	Maximum bactericidal effect on Gram-positive bacteria and *C. parapsilosis*.
	1:0.1	100.0	46.6	100.0	100.0	
Ch:HDTPPBr	1:0.012	100.0	29.3	20.2	100.0	
	1:0.1	100.0	28.2	35.5	100.0	
	1:0.5	100.0	48.6	40.4	100.0	

**Table 6:** Structure of sulfur extractants.

2-mercaptobenzothiazole (MBT) C_7_H_5_NS_2_	Thiourea (THIO) CH_4_N_2_S
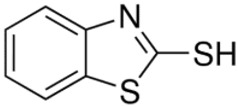 [77]	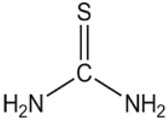 [78]

**Table 7:** Inhibition rate of bacterial and fungal growth when using sulfur materials.

Material	Ratio Ch:Extractant	Inhibition Rate (%)			
		*S. aureus* ATCC 25923	*P. aeruginosa* ATCC 27853	*E. coli* ATCC 25922	*C. parapsilosis* ATCC 22019
Ch:MBT	1:0.012	38.6	22.8	25.8	80.6
	1:0.1	64.0	26.3	33.6	83.6
	1:0.3	100.0	33.5	70.3	100.0
	1:0.5	100.0	38.8	72.3	100.0
Ch:Thiourea	1:0.012	43.5	36.6	38.6	81.5
	1:0.1	48.3	38.6	43.5	86.7
	1:0.5	54.4	46.7	51.0	100.0

## Data Availability

Not applicable.
